# Experimental and Numerical Study on Perforated Plate Mitigation Capacity to Near-Field Blasts

**DOI:** 10.3390/ma16124255

**Published:** 2023-06-08

**Authors:** Constantin-Cristinel Puică, Eugen Trană, Cristina Pupăză, Petrică Turtoi, Adrian-Nicolae Rotariu, Iuliana-Florina Pană

**Affiliations:** 1 Military Equipment and Technologies Research Agency, 16 Aeroportului, 077025 Clinceni, Romania; cpuica@acttm.ro; 2 Military Technical Academy ‘Ferdinand I’, 39-49 George Coșbuc Avenue, 050141 Bucharest, Romania; adrian.rotariu@mta.ro (A.-N.R.); florina.pana@mta.ro (I.-F.P.); 3 Doctoral School of Industrial Engineering and Robotics, University Politehnica of Bucharest, 313 Splaiul Independenței, 060042 Bucharest, Romania; cristina.pupaza@upb.ro; 4 Department of Machine Elements and Tribology, University Politehnica of Bucharest, 313 Splaiul Independenței, 060042 Bucharest, Romania; petrica.turtoi@upb.ro

**Keywords:** shock wave, perforated plate, passive mitigation, numerical study, impulse

## Abstract

Based on the analysis of existing collective shockwave protection methods worldwide, this paper addresses the mitigation of shock waves by means of passive methods, namely the use of perforated plates. Employing specialized software for numerical analysis, such as ANSYS-AUTODYN 2022R1^®^, the interaction of shock waves with a protection structure has been studied. By using this cost-free approach, several configurations with different opening ratios were investigated, pointing out the peculiarities of the real phenomenon. The FEM-based numerical model was calibrated by employing live explosive tests. The experimental assessments were performed for two configurations, with and without a perforated plate. The numerical results were expressed in terms of force acting on an armor plate placed behind a perforated plate at a relevant distance for ballistic protection in engineering applications. By investigating the force/impulse acting on a witness plate instead of the pressure measured at a single point, a realistic scenario can be considered. For the total impulse attenuation factor, the numerical results suggest a power law dependence, with the opening ratio as a variable.

## 1. Introduction

Over the last decades, a significant increase in terrorist attacks and asymmetric conflicts has been observed worldwide. In all these situations, the use of energetic materials (low or high explosives) against civil or military infrastructure (buildings or/and civil or military vehicles) is basically something common. Since such a scenario often results in material loss and human casualties, efforts have been made to increase protection against such threats. In line with all these efforts are the numerous studies regarding blast wave mitigation.

Blast-wave mitigation can be achieved through several methods that can be classified based on the principle of the method as: active (the system detects the threat and deploys the mitigation system) or passive (no activation mechanism is required).

Passive shock wave mitigation techniques include mechanisms designed to absorb, break and ultimately reduce the front pressure of the shock wave before it reaches the target. Usually, the costs associated with this type of protective structure are less significant than those based on the active approach.

Generally, passive mitigation systems are based on:

I. Impedance mismatch: placing a water cavity [1] or porous materials [2,3,4,5,6,7,8] in front of the target structure.
II. Composite structure implementations are structures employed to absorb energy through plastic deformation, limiting the transferred force due to the specific stress-strain relationship of the material. Significant examples are hyperelastic materials [9,10], metal foams [11,12,13] and sandwich structures [14].
III. Detonation wave deflection: systems that deflect the blast away from the structure they are protecting, such as V-shells [15,16,17].
IV. Shock wavefront braking: structures (perforated plates or a 3D architecture) designed to disrupt the shock wave front, placed between the detonation wave source and the target [18,19,20,21,22,23,24,25,26,27].

The design of passive blast wave attenuation systems has been a concern of scientists since the 1950s. As a result, numerous papers have been published in which the problem of blast wave mitigation has been addressed by both theoretical and experimental means.

On the narrower topic of blast wave mitigation using perforated plates, the theoretical approach was based on the fact that when the shock wave interacts with an obstacle, two processes occur simultaneously. On one hand, the shock wave is reflected by the obstacle, a phenomenon called shock wave reflection, and on the other hand, the wave is transmitted forward, generating an unsteady and turbulent flow behind the obstacle. From a theoretical perspective, more details regarding the interaction between the detonation wave and a perforated plate are presented in the work of Chao and Lee [28] and Britain et al. [21].

A common approach to the topic is the experimental investigation, which is essentially based on the employment of one of the two widely accepted test setups, namely the shock tube (compression-driven or explosive-driven) or live explosive tests on real or scaled models.

Although the experimental approach seems to be more straightforward, the costs and personnel safety challenges associated with this option should be carefully assessed, especially when considering full-scale testing. In fact, these are the main reasons to consider a numerical or hybrid experimental/numerical evaluation of such applications as a viable alternative, at least at the predesign stage.

The ability of single and multiple layers of perforated plates to attenuate blast overpressure and impulse was investigated both for close ducts (equivalent to venting ducts in buildings) [20,21,23,27] and open volumes (equivalent to explosive detonation beneath or on the side of a vehicle) [26]. Essentially, all of these studies were carried out using shock tubes. Although the use of shock tubes when investigating the blast wave mitigation capability of the perforated plates is extremely beneficial, the conditions reproduced in such tests can greatly differ from the real ones, especially when analyzing near-field explosions. The main differences are related to the fact that, in the case of shock tube tests, the shock wave front is planar and covers the entire surface of the perforated plate. In the case of near-field blast events, this is not necessarily true. 

To summarize, the ultimate goal of blast wave mitigation is to attenuate the blast wave and thereby significantly reduce its effect. Since the blast wave effect is correlated with the blast wave front pressure (both static and dynamic) and its action time, it is appropriate to say that blast mitigation is strongly correlated with the impulse analysis and sometimes, depending on the discussed application, with a pressure threshold.

**Figure 1:**
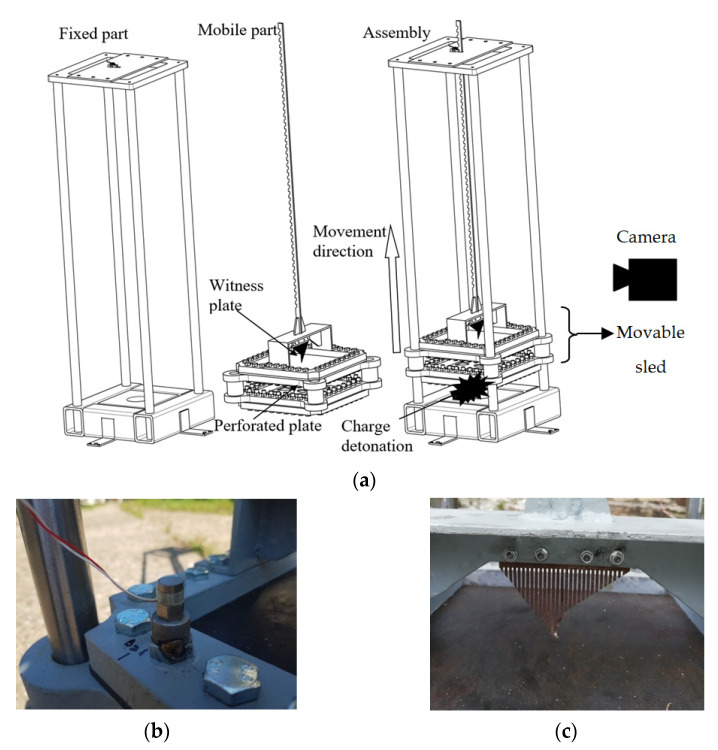
Experimental set-up (**a**) general view, (**b**) piezoelectric acceleration sensor position and (**c**) comb-type device position).

The main objective of the present paper is to numerically study the ability of a single perforated plate layer to reduce the effects of shock waves generated by near-field blasts. The shock wave attenuation capability of the perforated plate included the analysis of the shock wave propagation mode in the interaction with the witness plate and the behavior of the witness plate following the dynamic loading. Therefore, an experimental set-up has been configured to calibrate the numerical model (built in ANSYS Autodyn^®^), which is subsequently employed to evaluate the blast wave attenuation of the single perforated plate layer with different opening ratios (the ratio of the perforated area to the plate area). The calibration of the numerical model was based on the residual deformation of the perforated plate and the Z-moment of the movable sled (the moment on the Z axis in the movement direction, as indicated in Figure 1). Employing the calibrated numerical model, several cases with different opening ratios for the perforated plate were investigated in terms of force and impulse acting on the witness plate. A comparison with the data available in the literature was carried out, and the peculiarities of the numerical model results were discussed.

## 2. Materials and Methods

### 2.1. Experimental Set-Up

Attenuation performance investigation of passive mitigation systems for real-scale equipment is an expensive experimental activity. Therefore, limiting the number of tests, scaling the samples and coupling the experimental approach with a numerical one can lead to a more effective testing program. Since the main goal of the paper is to instrument the blast wave attenuation by a single perforated plate layer with different opening ratios in a near-field blast scenario, a custom experimental test rig was designed using a scaling factor of 5.5. The experimental setup was targeted to allow the calibration of the numerical model. The experimental setup is depicted in Figure 1.

The following measuring instruments and materials were included in the experimental setup:

(a) PCB piezotronics, model M350B23, piezoelectric acceleration sensor with ICP (integrated circuit piezoelectric). Using a filleted part, the sensor was fixed to the movable part of the device (Figure 1b). Its main purpose was to enable the recording of the movable part velocity when interacting with the blast wave;
(b) Comb-type device located behind the upper plate as presented in Figure 1c. The device was employed to evaluate the displacement of the witness plate;
(c) Photron Fastcam SA 1.1 high-speed video camera. Using a setup of 15,000 fps (i.e., frame per second) at a resolution of 320 × 1024 pixels, the global phenomenon and mobile sled (the mobile part) displacement as a function of time were recorded;
(d) Steel perforated plate (310 mm × 310 mm × 2.62 mm) with round perforations (ϕ = 40 mm) fixed to the movable frame by means of 36 M16 screws (Figure 1);
(e) A steel witness plate (5.92 mm thick) placed 90 mm above the perforated plate is also fixed to the movable frame by means of 36 M16 screws;
(f) Thirty-one-gram mass cylinder charges of HITEX^®^ (equivalent to C4) as a surrogate charge placed in a steel pot were used to produce a blast wave when detonated at 92 mm beneath the perforated plate and 182 mm beneath the witness plate. The HITEX^®^ explosive mass (with an TNT equivalency of 1.15 on impulse [29]) and the stand-off distance were adopted to ensure the same condition (the same value for the scaled distance parameter Z = 0.55 m/kg^1/3^) as in the case of in-soil buried 6 kg of TNT (Level 2b protection as defined by STANAG 4569) placed at approximately 1 m beneath a vehicle belly.

During the tests, two scenarios were investigated (Table 1). The main difference between the two scenarios refers to the presence of the perforated plate.

### 2.2. Numerical Model Set-Up

Complex phenomena are difficult and expensive to investigate, even when modern equipment is involved. The blast wave interactions with the perforated plate structures fall into the category of highly complex phenomena due to the speed and the number of parameters that directly impact the final results. This is even more complicated when the blast front gradually interacts with an obstacle.

Since time and cost control have always been concerns in engineering applications, in recent decades the numerical codes have been continuously improved to the point where the numerical simulation is starting to be a very useful design and analysis tool, especially in the early stages of the product development. ANSYS-AUTODYN 2022R1^®^ is a specialized software that uses the finite element method (FEM) and a range of numerical techniques to optimize, among other things, the analysis of equations describing nonlinear dynamic problems. 

The numerical investigation of blast waves is widely used nowadays in order to investigate several key parameters, such as pressure-time profile [30] or the propagation mode in various mediums [31]. 

**Figure 2:**
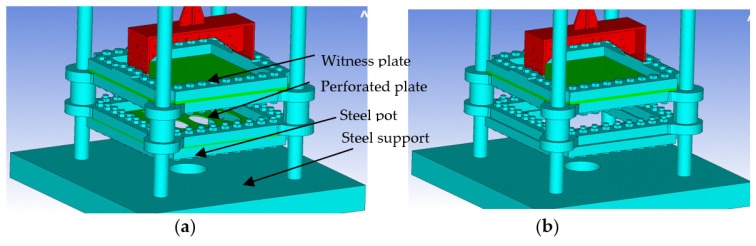
Studied configuration: (**a**) test with perforated plate (**b**) test without perforated plate.

As mentioned in Section 1, in the present research, the shock wave attenuation ability of a perforated plate will be investigated for the near-field blast scenario. The main objective is to examine the shock wave front propagation and the transmission through the perforated plate to the witness plate. The defined numerical model is illustrated in Figure 2.

In order to reduce the postprocessing time and the computing power without affecting the accuracy of the results, the REMAP option was considered in the numerical model computation up to the moment of interaction between the perforated plate and the shock wave. Basically, by using the REMAP option, the state variables computed on a 2D mesh were transferred over a pseudo-time interval to a new 3D mesh. In order to use the REMAP option for a mixed, Lagrange/Euler, multimaterial computational domain, two steps were completed:

- running a 2D axial-symmetric model of the explosion until the shock wave front is near the plate;
- initialization of the 3D plane-symmetric model of the explosion by loading the data obtained in the 2D axial-symmetric computation, using the REMAP functionality; in this case, the processing can be performed in parallel.

**Figure 3:**
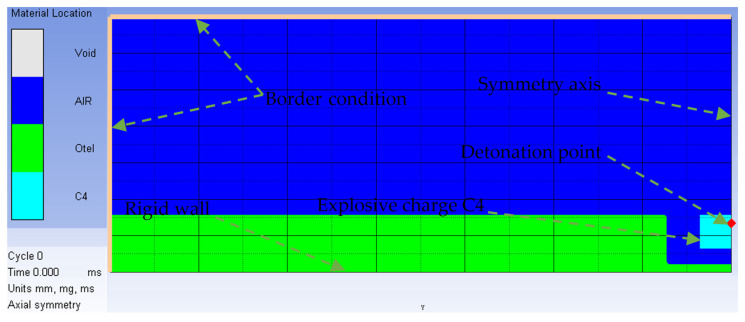
Two-dimensional axial-symmetric model.

Since the accuracy of FEM numerical model results is mesh size dependent, using a 2D axial-symmetric model of 0.25 × 0.25 mm cell dimension ensures high precision of the results in a reasonable amount of time, which cannot be achieved in a 3D approach. The initial 2D model and the included boundary conditions are depicted in Figure 3. The imposed flow-out condition (as boundary condition) aims to simulate the transient nature of the phenomenon (the explosive products are able to escape from the model, so a perfect uniform pressure state cannot be achieved).

**Figure 4:**
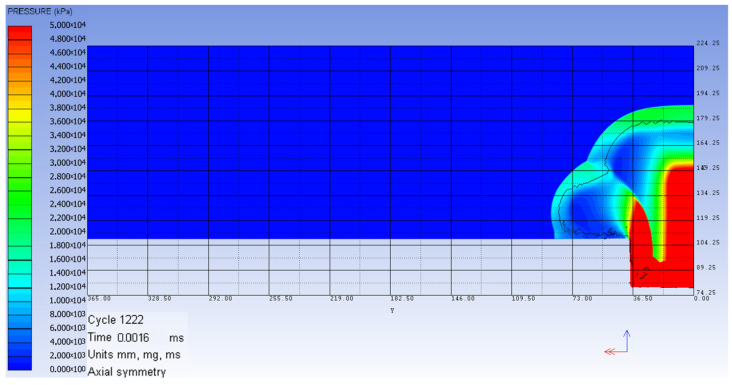
Numerical 2D model at 0.016 ms from detonation.

As mentioned before, the 2D axial-symmetric model was computed only up to the moment when the shock wave front arrived in the vicinity of the metallic structure, but without interacting. The obtained pressure fringe is presented in Figure 4. 

**Figure 5:**
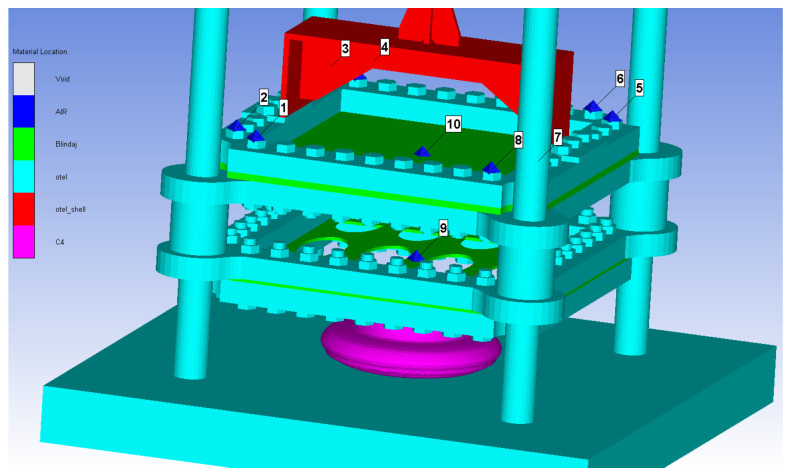
Numerical 3D model with detonation started and virtual sensor positions (Gauges 1 to 10).

The 3D model was setup by “rotating” the 2D model around the axis of symmetry when the shock wave front arrived in the vicinity of the metallic structure. Thus, the file created is loaded with data provided by the 2D axial-symmetric simulation and adapted for the 3D model (Figure 5).

In order to calibrate the numerical model, two scenarios (Table 1) were considered and simulated:

(a) The explosive charge detonation takes place in the presence of both the witness plate and the perforated plate (both composed of the same material—ARMOX 500).
(b) The explosive charge detonation occurs only in the presence of the witness plate (no perforated plate is present).

To simulate the previously mentioned scenarios:

- the detonating charge (explosive material) and the surrounding environment (air as an ideal gas) were modeled with the Euler–Gudonov algorithm. The algorithm refers to a conservative numerical scheme for solving partial differential equations (Euler equations), namely Gudunov’s scheme, assigned to the detonating charge and the surrounding environment;
- the boundary condition was set to a flow-out condition, allowing gases to be vented from the computation domain;
- the explosive was modeled with the Jones–Wilkins–Lee (JWL) equation of state. JWL is a Mie–Grueneisen form equation of state with a constant Grueneisen coefficient and a constant specific heat, widely employed to model the products of high explosives. The coefficients were adopted from Autodyn library for C4;
- the metal armor plates were modeled with Mie–Gruneisen equation of state and Johnson–Cook constitutive model. Model coefficients were adopted from [32].

**Figure 6:**
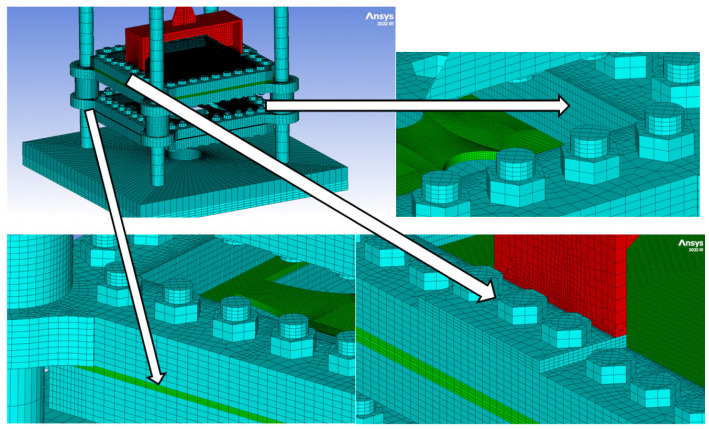
Numerical 3D model with conformal nodes.

To solve the numerical simulation in parallel using the interaction type-external gap, the model was built using conformal nodes (nodes from different parts that are in contact must coincide). Unfortunately, this particular approach generates a very large number of elements (Figure 6). For the simulation, 8 hexahedral node elements were used, and since all the nodes in contact were considered coincident, bolt pretension was not taken into account.

To optimize the computational resources, the simulations were carried out in two stages. In the first stage, the explosive charge placed in a steel support was detonated, and the expanded gases interacted with the plates. The duration of the simulated phenomenon in stage 1 was 1.89 ms. After 1.89 ms, the air and the explosive were removed from the numerical model and the simulation continued until the plate oscillations were damped, so that the residual deformations of the perforated and of the witness plate were identified. The damping ratio used in the analysis starting from t = 1.89 ms was 7.5·10^−6^.

The model was solved in parallel with 64 cores on a system consisting of 2 computing nodes, connected by Infiniband EDR 100 Gb, with two Intel Xeon 6140 18-core processors, 2.3 GHz, 384 Gb RAM DDR4 2666 ECC each, and lasted 1350 h.

**Figure 7:**
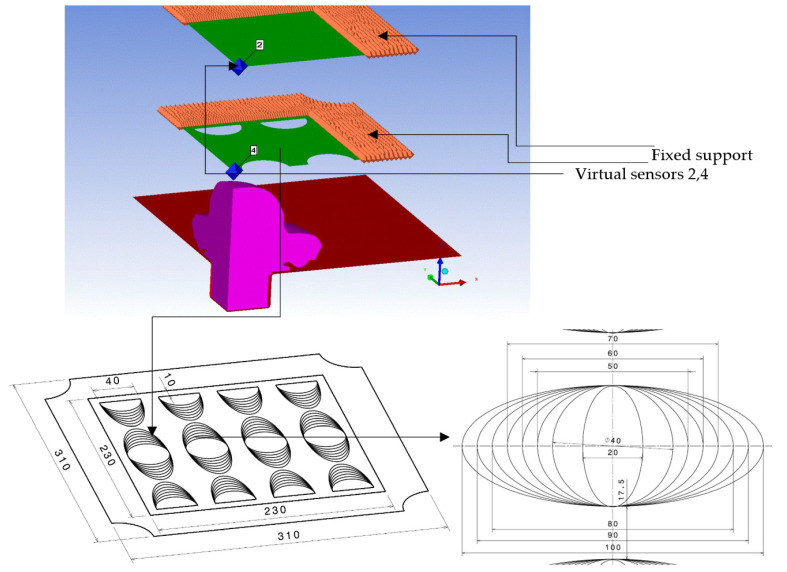
Setup of numerical simulations on the simplified quarter model with two gauges.

Once the numerical model calibration was achieved, seven other numerical simulations were performed for different opening ratios. As processing resources and time are both important and limited, the investigated cases were simplified to a quarter model (Figure 7).

Figure 7 depicts the elliptic-shaped perforations of the perforated plate, ensuring an opening ratio variation between 10% and 50% (Table 2). In order to study only the influence of the perforated plate on the impulse transfer, the rigid connection between the two plates (the movable part) was removed and the two plates were fixed. 

## 3. Results

**Figure 8:**
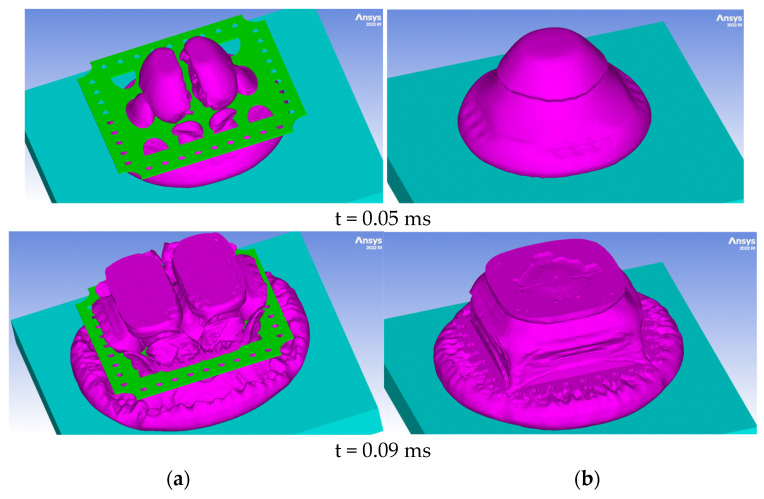
Evolution of the explosion reaction products (**a**) with perforated plate (**b**) without perforated plate.

Figure 8 shows the computed shock wave front shape for two different time moments (t = 0.05 ms and t = 0.09 ms) for both simulated scenarios. As can be seen in Figure 8a, the presence of the perforated plate results in a shock wave front disruption. The interaction forms a turbulent and nonstationary flow.

**Figure 9:**
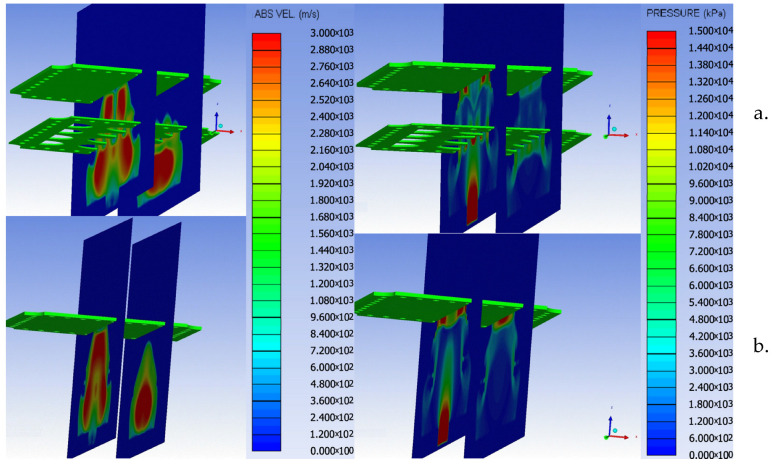
Pressure and velocity distribution on the witness plate with (**a**) and without (**b**) perforated plates at different moments.

The pressure and velocity fringes (Figure 9) for the two simulated scenarios clearly exemplify the shock wave-breaking phenomenon in the presence of the perforated plate.

**Figure 10:**
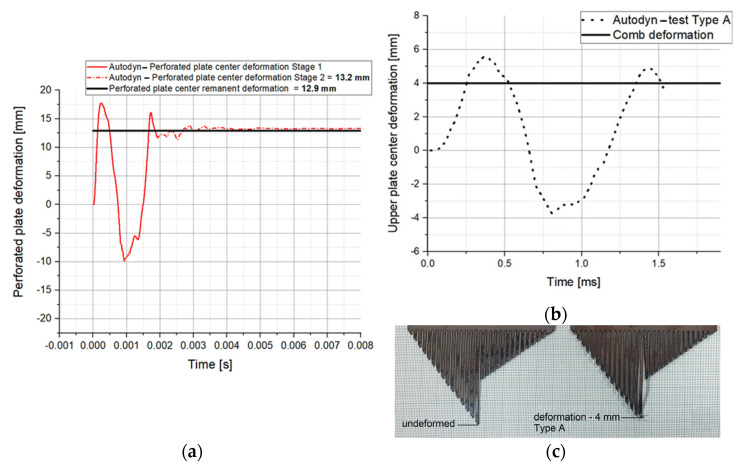
The recorded deformation in type A test (**a**) residual deformation of perforated plate, (**b**) displacement of witness plate and (**c**) comb-type device deformation.

Figure 10 plots the post-test experimental measurements against time according to the numerical ones in terms of the perforated plate deflection and the witness plate. 

The residual deformation of the perforated plate in the simulation is 3% higher than the one obtained in the type A tests.

**Figure 11:**
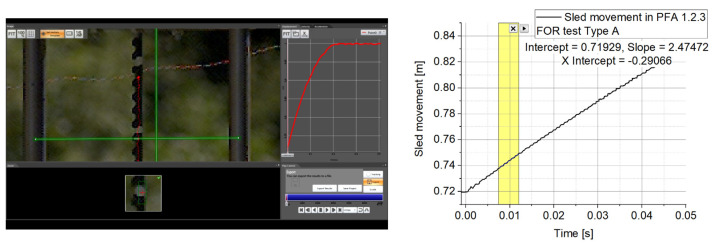
Determination of initial sled speed in Type A tests.

Since the numerical model calibration targets were both the perforated plate deflection and the witness plate Z-momentum, in Figure 11, the camera-recorded data is displayed. 

The Photron Fastcam SA 1.1 high-speed camera recordings were processed in terms of the movable sled initial speed with the help of the Photron Fastcam Analyzer program (Figure 11).

**Figure 12:**
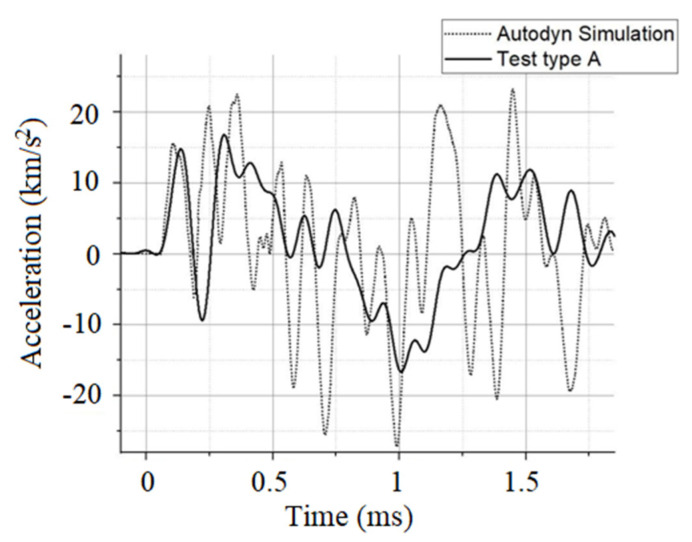
Acceleration comparison (Type A tests).

The PCB sensor recorded data plotted against the numerical simulation results are depicted in Figure 12 in terms of sled acceleration for the Type A case.

**Figure 13:**
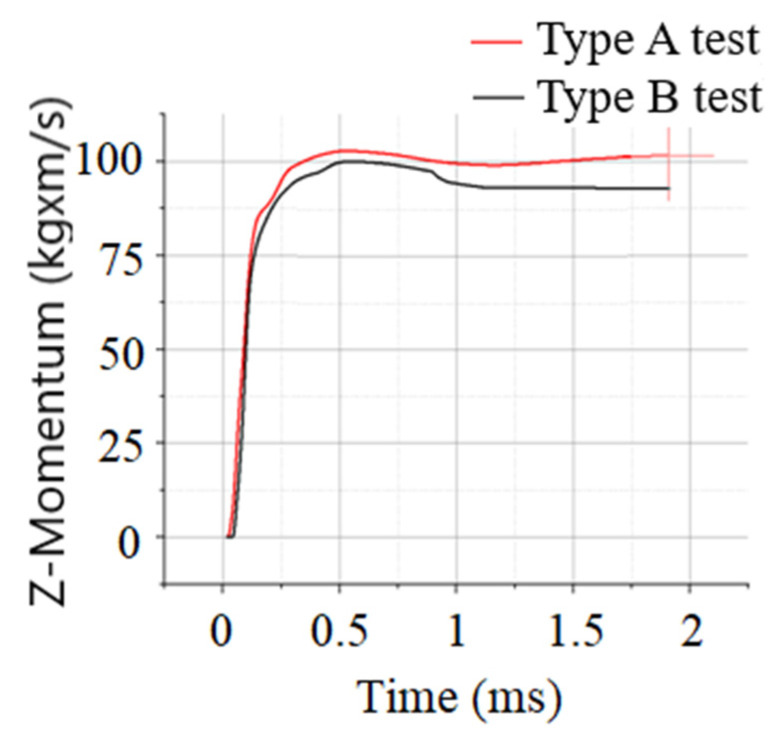
Numerical results for sled Z-momentum vs. time.

Z-momentum results for the numerical model calibration tests are presented in Figure 13.

A synthesis of the most relevant data regarding the witness plate impulse as part of the numerical model calibration process is recorded in Table 3. 

## 4. Discussion

Military vehicle protection against blast wave events in security-challenging areas is a top priority. Overpressure and impulse mitigation are the main goals in the design of both active and passive protection systems/structures. The configuration of the structure to be protected is of great importance in the design of the protective devices, and usually the real field conditions are hard to reproduce within simple laboratory tests. 

When discussing blast wave protection, the greatest challenge is raised by the already in-service vehicles, where protection can be achieved only by considering the use of additional mitigation systems. Placing perforated plates beneath the military vehicle’s belly is a costless and possibly effective approach [31,32,33] to retrofit an already in-service design to withstand higher blast wave loads. 

The proposed numerical approach has some limitations that are very likely to be present when considering the real design cases of the add-on mitigation systems. Thus, ground clearance and mass, as key parameters for vehicle mobility, especially in an off-road scenario, severely limit the distance that can be achieved between the explosive charge and the perforated plate, as well as the distance between the perforated plate and the witness plate. Thereby, the number of perforated plate layers that can be considered is also limited, even though, as pointed out in [26,27], the number of perforated plate layers can increase the attenuation factor (defined as the ratio of overpressure acting on the witness plate without and in the presence of the perforated plate). As a result, the analyses performed for a single perforated plate layer, with 90 mm clearance between the two plates and 92 mm between the explosive charge and the perforated plate, closely simulate a real IED (improvised explosive device) or mine attack scenario. Due to the close detonation range and the fact that the explosive charge was placed in a steel pot for both the experimental tests and the numerical simulations (simulating the metallic envelope and semiburied nature of an IED/mine), it can be safely stated that the study refers to a highly probable near-field blast scenario.

The decision to choose round/elliptical perforations for both the experimental test and the numerical simulation was based on the results detailed in [20,24,26,27], which state that for a fixed ratio between the distance at which the attenuation is evaluated and the smallest dimension of the perforations, the opening ratio is the predominant factor in the attenuation process, while the shape and number of perforations play an insignificant role.

The values adopted for the opening ratio in the numerical simulations are in the range of 0.1–0.5. Values less than 0.1 were ignored due to the significant mass addition involved. They are less likely to be adopted in a practical configuration. Values greater than 0.5 are also unlikely because, as concluded in [23], such large openings have a minor or no effect in terms of mitigating the witness plate deflection.

**Figure 14:**
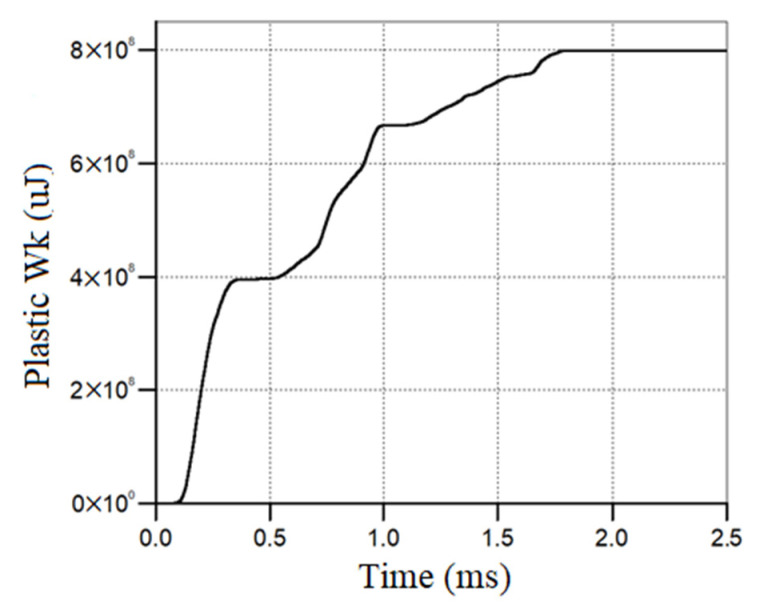
Perforated plate plastic work vs. time.

The calibration method involved two key parameters: the perforated plate deflection and the sled impulse, respectively. The first one ensures mainly the material model validity, while the second one is focused on the global phenomenon. As depicted in Figure 10, the numerical results and the experimental ones are in good agreement in terms of the residual deformation, but not so good in terms of the total deformation, where the numerical simulation overrates the parameter. Regarding the time moment that was chosen to remove the air and the explosive gas products from the numerical model, it must be stated that it was based on the plastic work’s calculated values. Figure 14 emphasizes that after 1.8 ms, no additional plastic work is present in the perforated plate.

As pointed out by the numerical simulations (Figure 9 is a representative example of such numerical results), in the presence of the perforated plate, the pressure field acting on the witness plate is irregular. The existence of those pressure variations makes the attenuation factor computation seem a little far-fetched when it is based on the pressure history data recorded at just one point on the witness plate surface. In order to take into account all the point contributions, the attenuation factor was investigated in terms of impulse (area beneath the force vs. time graph) acting on the witness plate surface. By employing this particular approach, the pressure profile has been leveled and the calibration method for the numerical model (based on the impulse evaluation) is more consistent. Furthermore, by evaluating the impulse, the complex phenomenon of pressure reflection can also be taken into account. 

**Figure 15:**
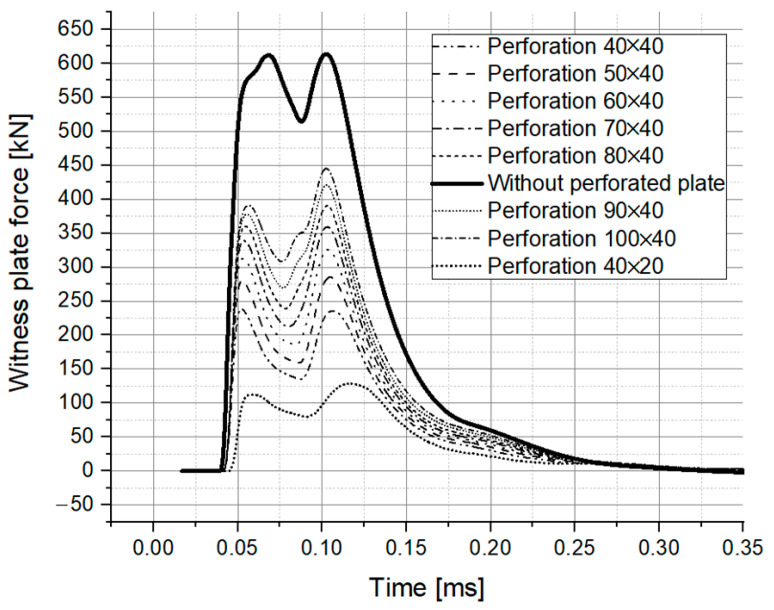
Force on witness plate vs. time.

The numerically tested configurations, as mentioned before, were based on an elliptical-shaped perforation. The results of the numerical simulations for all nine scenarios are depicted in Figure 15 in terms of the force acting on the witness plate. 

Analyzing Figure 15, it can be observed that for all the tests, a double hump profile for the force acting on the witness plate is recorded. The solid line in Figure 15 corresponds to a type B test, meaning that the recorded values correspond to the case in which the perforated plate is missing. Basically, these are the values that are targeted to be decreased by placing the perforated plate in front of the witness plate.

Analyzing the numerical simulation results for the type B tests in a step-by-step approach, it was observed that the first hump is due to the incident blast. After reaching the maximum value, due to the gas expansion, the force acting on the witness plate starts to decrease. This trend is, however, inverted by a second shock wave emerging from the steel pot, generating a second hump. Even if it is not clear, the second shock could be explained as the result of a complex interaction of the pressure waves inside the steel pot, which, due to its side walls, alters the flow of the gas products. 

The same double hump profile can also be observed for the type A tests. However, there are some clear differences compared with the test B results. Thus, Figure 15 undoubtedly depicts that with the decrease in the opening area, the force on the witness plate decreases, both for the first hump, as well as for the second one. Even more, it can be observed that the second peak shifts in time. The shift could be explained by the correlation between blast wave intensity and its velocity. As a particularity of the type A tests, it can be noted that the second peak is slightly higher compared with the first one due to the presence of the perforated plate.

**Figure 16:**
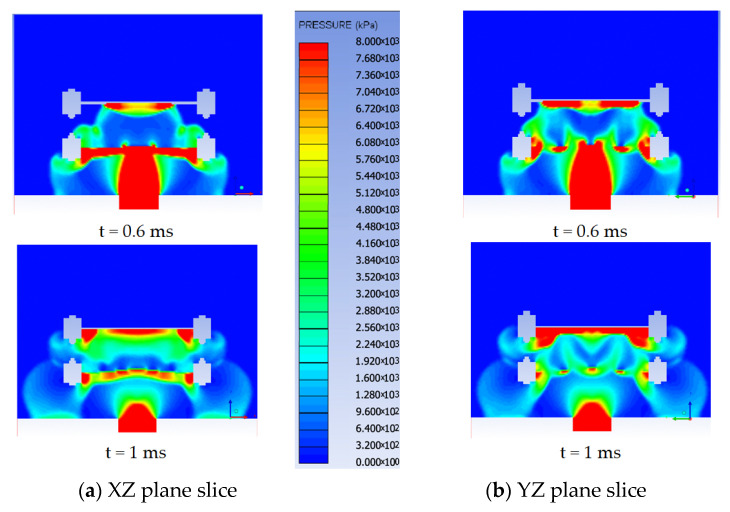
Blast wave acting on the witness plate for Type A test.

Another observed peculiarity is that, due to the perforation dimensions and the clearance between the explosive charge and the perforated plate, the shock wave front gradually expands itself to cover the entire surface of the witness plate (Figure 16).

The gradual expansion of the blast wave on the witness plate and the low clearance is, in fact, an important deviation from the regular shock tube test situation. Having two humps on the graph poses some difficulties in evaluating the attenuation factor that the perforated plate provides for the near-field blast events. A possible way to get around this issue is to approach the attenuation factor concept in two different ways. Firstly, by evaluating the impulse ratio (the impulse calculated as the area beneath the force vs. time curve) for the first hump (up to the moment when the force starts to increase again), one can evaluate the impulse attenuation for the incident shock wave front (similar to the case of the shock wave tube tests). Secondly, by evaluating the impulse ratio for both humps (the entire area beneath the force vs. time curve), both the first and second shock waves can be considered, along with the gradual action of the explosive gas products on the witness plate. The results obtained for the first hump (partial impulse) considering an opening ratio of 0.48 are in agreement with the results previously reported by Schunck et al. [26] for large and small round perforations (less than 5% deviation). 

**Figure 17:**
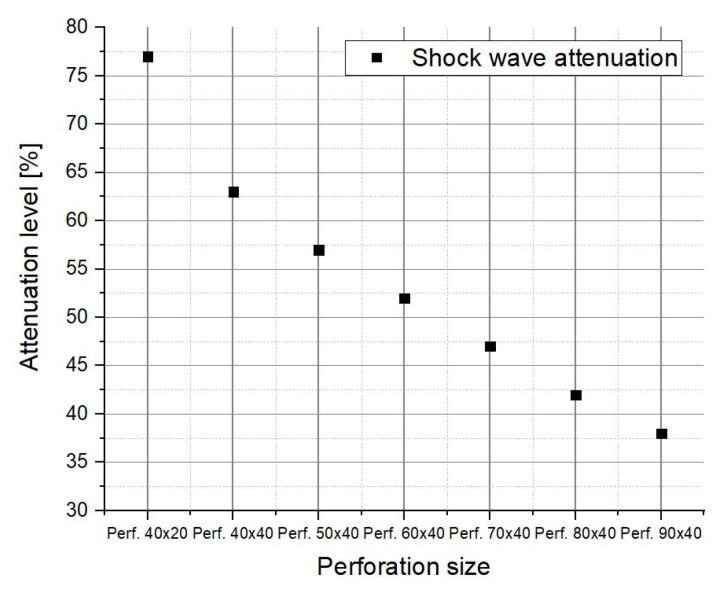
Total impulse attenuation level.

Considering the total value of the impulse (both humps), the attenuation factor dramatically decreases (Figure 17), emphasizing that the presence of a secondary blast wave can severely affect the perforated plate’s performance. 

**Figure 18:**
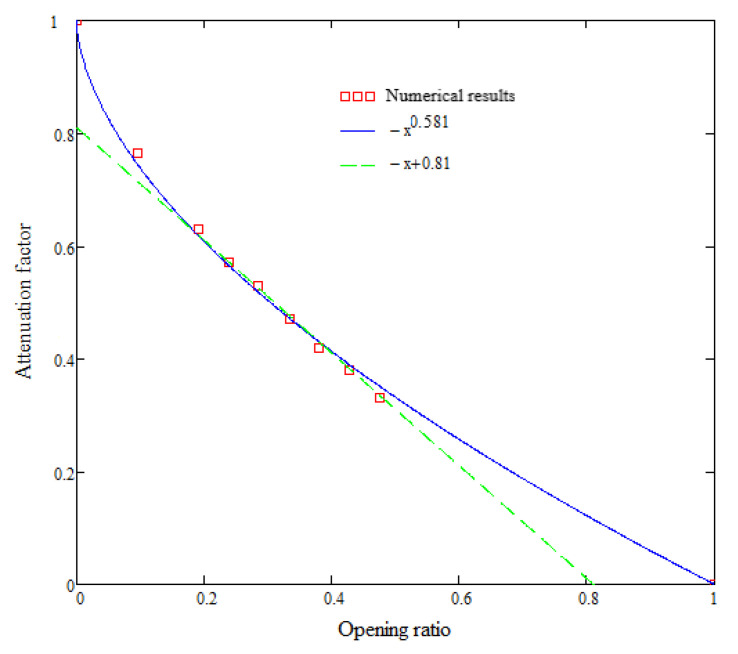
Impulse attenuation factor function.

Including both range ends, in Figure 18, the attenuation factor plot for the total impulse can be expressed as a power function (the solid line), somehow similar to the one reported in [18] for the pressure. If the attenuation is discussed for a narrow interval of the opening ratio, 0.2–0.5, the attenuation factor can be easily approximated as a linear function (dashed line) (Figure 18).

## 5. Conclusions

The present paper is a numerical study of the ability of perforated plates to mitigate blast waves. The investigated scenario refers to a near-field blast event that closely simulates a shallow-buried explosive detonation. The numerical computation allows a detailed investigation of the phenomenon, even though it cannot supersede the experimental investigation. The numerical results of the near-field blast scenario point out that the perforated plate and the witness plate are loaded by not just a single shock wave front but by two of them. The numerical investigation highlighted that the near-field blast wave does not interact with the entire plate surface all at once, as in this case, and under the conditions imposed by shock wave blast tube tests. As expected, the impulse mitigation is strongly related to the opening ratio of the perforated plate. The dependence of the attenuation factor on the opening ratio can be approximated as a power law. However, for narrow opening ratios (0.2–0.5), the linear dependence may prove to be a much easier and faster method. For the real vehicle configuration, the assessment of the mitigation capability of the perforated plate must also take into account aspects related to the impulse transmission between the perforated plate and the vehicle floor. 

## Figures and Tables

**Table 1:** Matrix of carried out tests.

Test Configuration	Witness Plate	Perforated Plate	Number of Tests
Type A with perforated plate	5.92 mm—Armox 500	2.62 mm—Armox 500	3
Type B without perforated plate	5.92 mm—Armox 500	-	3

**Table 2:** Opening ratio.

Perforation dimensions (mm)	40 × 20	40 × 40	40 × 50	40 × 60	40 × 70	40 × 80	40 × 90	40 × 100
Opening ratio	0.095	0.190	0.238	0.285	0.333	0.380	0.428	0.475

**Table 3:** Comparison of impulse and velocity of mobile sleds.

Numerical Simulation/Test	Mass [Kg]	Z-Momentum [kgxm/s]	Velocity [m/s]	Z-Momentum Comparison [%]	Velocity Comparison [%]
Autodyn Type A tests	40.48	101.60	2.51	5.61	1.61
Autodyn Type B tests	39.04	92.52	2.37	8.18	3.94
Experimental Type A tests	38.95	96.20	2.47	-	-
Experimental Type B tests	37.51	85.52	2.28	-	-

## Data Availability

The raw/processed data required to reproduce these findings cannot be shared at this time, as the data also forms part of an ongoing study.

## References

[1] Absil L., Bryntse A. (2006). Blast Mitigation by Water.

[2] Ben-Dor G., Britan A., Elperin T., Igra O., Jiang J.P. (1997). Experimental investigation of the interaction between weak shock waves and granular layers. Exp. Fluids.

[3] Rotariu A., Trană E., Dima C., Enache C., Timplaru F., Matache L. (2016). Uninstrumented measurement method for granular porous media blast mitigation assessment. Exp. Tech..

[4] Guéders C., Van Roey J., Gallant J., Coghe F. Simulation of Shock Wave Mitigation in Granular Materials by Pressure and Impulse Characterization. Proceedings of the 8th European LS-DYNA Users Conference.

[5] Grujicic M., Pandurangan B., Bell W.C., Bagheri S. (2012). Shock-Wave Attenuation and Energy-Dissipation Potential of Granular Materials. J. Mater. Eng. Perform..

[6] Britan A., Ben-Dor G., Igra O., Shapiro H. (2001). Shock waves attenuation by granular filters. Int. J. Multiph. Flow.

[7] Nesterenko V.F. (2002). Shock (Blast) Mitigation by ‘‘Soft’’ Condensed Matter. Proceedings of the Matter Research Society Symposium: Granular Materials-Based Technologies, Materials Research Society.

[8] Walley S.M., Proud W.G. A Comparison of the Quasistatic and Dynamic Compressibilities of Wet and Dry Vermiculite. Proceedings of the 9th International Conference on the Mechanical and Physical Behavior of Materials under Dynamic Loading.

[9] Bucur F., Rotariu A.N., Trană E. (2019). Numerical and Experimental Study on the Locally Blast Loaded Polyurea Coated Steel Plates. Mater. Plast..

[10] Bucur F., Trana E., Rotariu A., Gavrus A., Barbu C., Guines D. Experimental and numerical analysis concerning the behaviour of OL50 steel grade specimens coated with polyurea layer under dynamics loadings. Proceedings of the 11th International Conference on the Mechanical and Physical Behaviour of Materials under Dynamic Loading, EPJ Web of Conferences.

[11] Rotariu A., Trană E., Matache L., Cirmaci-Matei V.M., Sandu S., Moldoveanu C.E., Bucur F. (2021). Experimental study on the dynamic response of polyurethane/fly ash ceramic foam. Mater. Plast..

[12] Hanssen A.G., Enstock L., Langseth M. (2002). Close-Range Blast Loading of Aluminium Foam Panels. Int. J. Impact Eng..

[13] Ousji H., Belkassem B., Louar M.A., Reymen B., Martino J., Lecompte D., Pyl L., Vantomme J. (2017). Air-blast response of sacrificial cladding using low density foams: Experimental and analytical approach. Int. J. Mech. Sci..

[14] Arora H., Hooper P.A., Dear J.P., Abrate S., Castanié B., Rajapakse Y. (2013). Blast Loading of Sandwich Structures and Composite Tubes. Dynamic Failure of Composite and Sandwich Structures.

[15] Kumar P., Leblanc J., Stargel D.S., Shukla A. (2012). Effect of plate curvature on blast response of aluminum panels. Int. J. Impact Eng..

[16] Chung K.Y.S., Langdon G.S., Nurick G.N., Pickering E.G., Balden V.H. (2012). Response of Vshape plates to localized blast load: Experiments and numerical simulation. Int. J. Impact Eng..

[17] Bucur F., Rotariu A., Trană E., Ștefan A. (2020). Experimental and Numerical Study on the Mitigation Capability of Some Special Design Structures. Int. J. Mod. Manuf. Technol..

[18] Kingery C., Pearson R., Coulter G. (1977). Shock Wave Attenuation by Perforated Plates with Various Hole Size.

[19] Nurick G.N., Martin J.B. (1989). Deformation of thin plates subjected to impulsive loading—A review part ii: Experimental studies. Int. J. Impact Eng..

[20] Britan A., Karpov A.V., Vasilev E.I., Igra O., Ben-Dor G., Shapiro E. (2004). Experimental and numerical study of shock wave interaction with perforated plates. J. Fluids Eng..

[21] Britan A., Igra O., Ben-Dor G., Shapiro H. (2006). Shock wave attenuation by grids and orifice plates. Shock. Waves.

[22] Langdon G.S., Nurick G.N., Balden V.S., Timmins R.B. (2008). Perforated plates as passive mitigation systems. Def. Sci. J..

[23] Langdon G.S., Rossiter I.B., Balden V.H., Nurick G.N. (2010). Performance of mild steel perforated plates as a blast wave mitigation technique: Experimental and numerical investigation. Int. J. Impact Eng..

[24] Langdon G.S., Nurick G.N., DU Plessis N.J. (2011). The influence of separation distance on the performance of perforated plates as a blast wave shielding technique. Eng. Struct..

[25] Berger S., Ben-Dor G., Sadot O. (2015). Experimental and numerical investigations of shock-wave attenuation by geometrical means: A single barrier configuration. Eur. J. Mech.-B/Fluids.

[26] Schunck T., Eckenfels D. (2021). Blast mitigation by perforated plates using an explosive driven shock tube: Study of geometry effects and plate numbers. SN Appl. Sci..

[27] Ram O., Ben-Dor G., Sadot O. (2018). On the pressure buildup behind an array of perforated plates impinged by a normal shock wave. Exp. Therm. Fluid Sci..

[28] Chao J., Lee J.H.S., Jiang Z. (2005). The interaction of a detonation with a perforated plate. Shock Waves.

[29] Bogosian D., Yokota M., Rigby S. TNT equivalence of C-4 and PE4: A review of traditional sources and recent data. Proceedings of the 24th Military Aspects of Blast and Shock Conference.

[30] Ivančo M., Erdélyiová R., Figuli L. (2019). Simulation of detonation and blast waves propagation. Transp. Res. Procedia.

[31] Tropin D., Temerbebkov V. (2022). Numerical simulation of detonation wave propagation through a rigid permeable barrier. Int. J. Hydrog. Energy.

[32] Kiliç N., Bedir S., Erdik A., Ekici B., Tașdemirici A., Guden M. (2014). Ballistic behavior of high hardness perforated armor plates against 7.62 mm armor piercing projectile. Mater. Des..

[33] Schunck T., Eckenfels D., Sinniger L. (2022). Blast disruption using 3D grids/perforated plates for vehicle protection. Def. Technol..

